# Diaphragmatic peritoneal metastases mimicking liver metastases

**DOI:** 10.1515/pp-2021-0120

**Published:** 2021-08-20

**Authors:** Barbara Noiret, Clarisse Eveno

**Affiliations:** Claude Huriez Hospital, Lille, France

**Keywords:** cytoreductive surgery, diaphragmatic peritoneal metastases, hyperthermic intraperitoneal chemotherapy (HIPEC), liver metastases, scalloping

**Figure 1:**
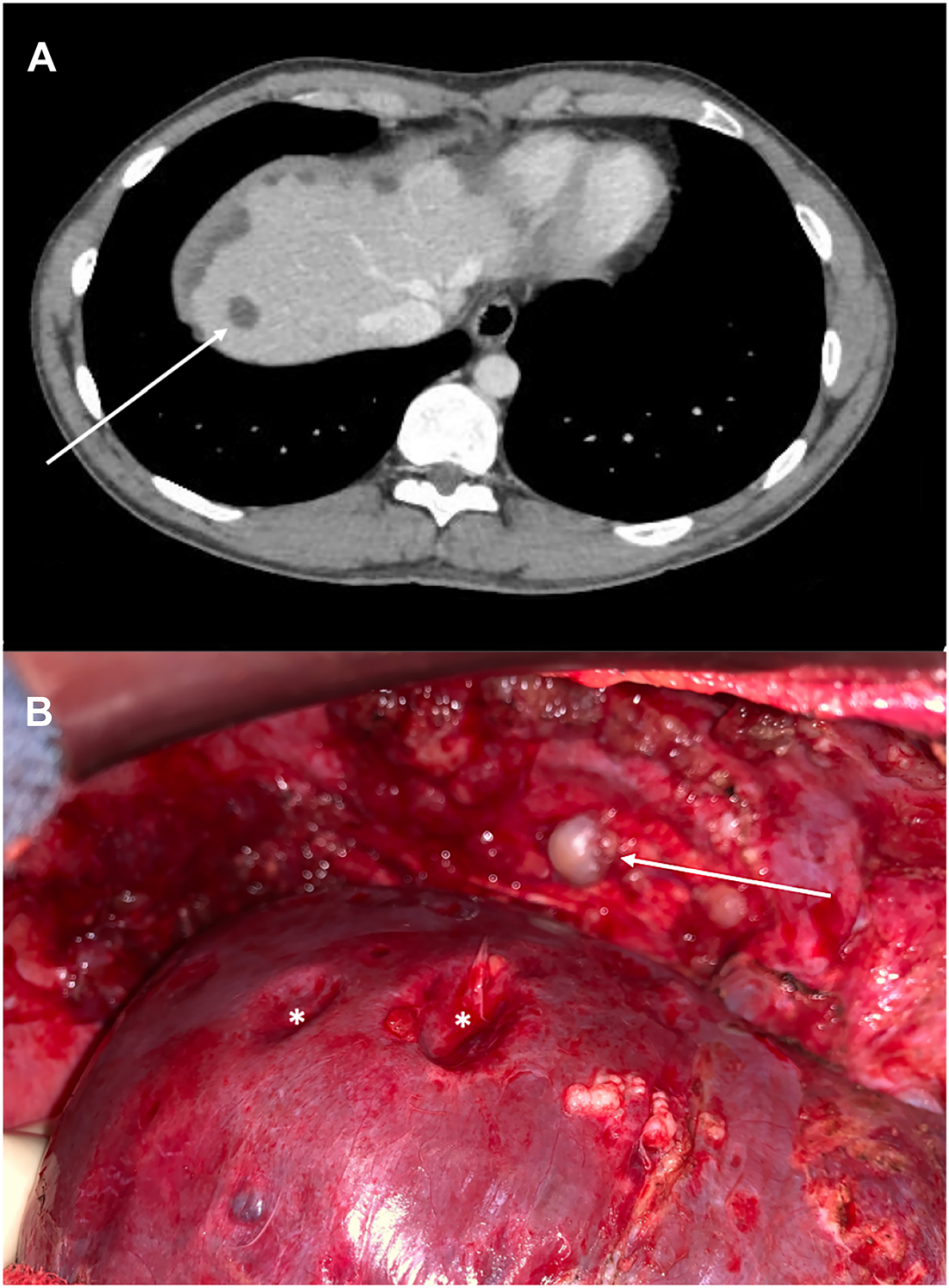
CT-scan (A) revealed hypodense liver lesions (arrow) at the diagnosis of left colon adenocarcinoma with synchronous peritoneal metastases. Explorative laparotomy (B) highlighted diaphragmatic peritoneal metastases (arrow) exerting pressure on the liver surface, called «scalloping» (star).

A 27-year-old male presented with abdominal pain and asthenia. Abdominal CT-scan identified left colon mass associated with synchronous peritoneal metastases (PM) and hypodense liver suspicious lesions (Figure 1A, arrow). Colonoscopy with pathological analysis of colic biopsies confirmed an adenocarcinoma with microsatellite instability, allowing to perform neoadjuvant immunotherapy (Pembrolizumab). Explorative laparotomy confirmed the PM with the presence of implants on the right diaphragmatic dome (Figure 1B, arrow) exerting extrinsic compression of the liver (Figure 1B, star). A complete cytoreductive surgery combined with Mitomycin C based-hyperthermic intraperitoneal chemotherapy (HIPEC) was performed.

PM are often located in Douglas pouch, parieto-colic gutters, and also in the subphrenic space. Therefore, peritoneal tumor implants under the diaphragm can mimic liver metastases on CT-scan [1, 2] by exerting pressure on the liver surface, called “scalloping” [3]. It is essential to distinguish diaphragmatic PM and liver metastases and to carry out further investigations in case of doubt, because of their different therapeutic strategies.
